# Usability Testing of a Reusable Pulse Oximeter Probe Developed for Health-Care Workers Caring for Children < 5 Years Old in Low-Resource Settings

**DOI:** 10.4269/ajtmh.18-0016

**Published:** 2018-08-20

**Authors:** Nicholas Boyd, Carina King, Isabeau A. Walker, Beatiwel Zadutsa, Mike Bernstein, Salahuddin Ahmed, Arunangshu Roy, Abu A.M. Hanif, Subal C. Saha, Kingshuk Majumder, Bejoy Nambiar, Tim Colbourn, Charles Makwenda, Abdullah H. Baqui, Iain Wilson, Eric D. McCollum

**Affiliations:** 1 Great Ormond Street Hospital, UCL Institute of Child Health, London, United Kingdom;; 2 Institute for Global Health, University College London, London, United Kingdom;; 3 Lifebox Foundation, London, United Kingdom;; 4 Parent and Child Health Initiative, Lilongwe, Malawi;; 5 Physio Monitor, LLC, San Ramon, California;; 6 Johns Hopkins University-Bangladesh, Dhaka, Bangladesh;; 7 Department of International Health, Johns Hopkins Bloomberg School of Public Health, Baltimore, Maryland;; 8 Eudowood Division of Pediatric Respiratory Sciences, Department of Pediatrics, Johns Hopkins School of Medicine, Baltimore, Maryland

## Abstract

Hypoxemia measured by pulse oximetry predicts child pneumonia mortality in low-resource settings (LRS). Existing pediatric oximeter probes are prohibitively expensive and/or difficult to use, limiting LRS implementation. Using a human-centered design, we developed a low-cost, reusable pediatric oximeter probe for LRS health-care workers (HCWs). Here, we report probe usability testing. Fifty-one HCWs from Malawi, Bangladesh, and the United Kingdom participated, and seven experts provided reference measurements. Health-care workers and experts measured the peripheral arterial oxyhemoglobin saturation (SpO_2_) independently in < 5 year olds. Health-care worker measurements were classed as successful if recorded in 5 minutes (or shorter) and physiologically appropriate for the child, using expert measurements as the reference. All expert measurements were considered successful if obtained in < 5 minutes. We analyzed the proportion of successful SpO_2_ measurements obtained in < 1, < 2, and < 5 minutes and used multivariable logistic regression to predict < 1 minute successful measurements. We conducted four testing rounds with probe modifications between rounds, and obtained 1,307 SpO_2_ readings. Overall, 67% (876) of measurements were successful and achieved in < 1 minute, 81% (1,059) < 2 minutes, and 90% (1,181) < 5 minutes. Compared with neonates, increasing age (infant adjusted odds ratio [aOR]; 1.87, 95% confidence interval [CI]: 1.16, 3.02; toddler aOR: 4.33, 95% CI: 2.36, 7.97; child aOR; 3.90, 95% CI: 1.73, 8.81) and being asleep versus being calm (aOR; 3.53, 95% CI: 1.89, 6.58), were associated with < 1 minute successful measurements. In conclusion, we designed a novel, reusable pediatric oximetry probe that was effectively used by LRS HCWs on children. This probe may be suitable for LRS implementation.

## INTRODUCTION

Pneumonia is the leading infectious cause of death in children < 5 years old, and an estimated 85% of all global pneumonia deaths occur in sub-Saharan Africa and South Asia.^1,2^ Severe pneumonia can be associated with hypoxemia, defined by the World Health Organization (WHO) as a peripheral arterial oxyhemoglobin saturation (SpO_2_) < 90%.^3^ Hypoxemia is strongly associated with child pneumonia mortality in low-resource settings (LRS) and can be detected noninvasively by pulse oximetry.^4^ Hospital oxygen systems using pulse oximetry in LRS are associated with reduced pneumonia mortality.^5^ Current WHO guidelines recommend the use of pulse oximetry at peripheral facilities only if available and provide no guidance for its use at the community level.^6,7^ However, it is widely recognized that routine pulse oximetry screening could improve pediatric pneumonia management in LRS.^8–10^

Routine pulse oximetry use for children in LRS has been limited despite the availability of high-quality, low-cost pulse oximeters designed for use in LRS, such as the Lifebox^®^ oximeter (New Taipei City, Taiwan).^9,11^ Lifebox^®^ Foundation is a nonprofit organization focused on safer surgery and anesthesia in LRS, and the foundation currently makes available a high-quality pulse oximeter and probe priced at $250 USD/unit. A recent pneumococcal vaccine effectiveness study demonstrated successful implementation and use of the Lifebox^®^ oximeter with an adult probe on > 13,000 children with clinical pneumonia between 2012 and 2014 across the routine health system in Malawi.^9,12^ However, the authors noted that SpO_2_ measurements were difficult to obtain in some children, particularly in younger infants and neonates, and that a low-cost, reusable probe designed specifically for children would significantly advance implementation of pulse oximetry in LRS.^9^ This project evolved from this key finding. In high-income settings single-use adhesive probes costing ∼$10 each are commonly used but are an unsustainable solution for LRS. A reusable probe, optimized for measuring SpO_2_ on children of all ages by health-care workers (HCWs) of varying training backgrounds would potentially be a key advancement for improving pneumonia care in LRS.

We used a human-centered design (HCD) approach^13^ that engaged end-users and experts from multiple disciplines into the development process of a reusable, low-cost pediatric oximeter probe. This study presents the summative usability testing process of our HCD approach. Our objective was to evaluate the usability of the probe by end-users and experts across a range of settings and children against an aspirational target product profile (TPP) goal of 95% of readings achieved within 1 minute (Supplemental Appendix 1). As HCWs in LRS are overburdened and have limited time per patient, we established this goal as an ideal time to obtain an SpO_2_ reading, based on inputs from experts and end-users. If achievable, this could optimize implementation feasibility of pulse oximetry in this setting.

## MATERIALS AND METHODS

We conducted usability testing of a novel pediatric pulse oximeter probe (LB-01), developed using HCD, using feedback from a modified Delphi technique to aid probe design refinements. The probe was used in combination with the Lifebox^®^ oximeter (version 1.5). Participants were HCWs in two LRS with a high pneumonia burden (Malawi and Bangladesh) and one high-resource setting (the United Kingdom [UK]), included as a site with highly trained HCWs. The research described in this article does not evaluate the device’s accuracy. Our study team (M. B.) evaluated the accuracy of this pulse oximetry probe separately in an in-vivo study at the University of California San Francisco. The device passed all testing according to pulse oximeter device regulatory standards.^14,15^

### Human-centered design with modified Delphi method.

The modified Delphi method is a series of consecutive investigations or rounds that seek organized, incremental feedback to achieve the most accurate views from experts.^16^ We incorporated this approach within our HCD process and stepwise usability testing, with end-users and experts providing feedback between each round. This allowed us to consider end-user–driven probe refinements before further testing.

### Settings.

#### Mchinji, Malawi.

Mchinji is located in central Malawi where health care is provided by community health workers (CHWs) called Health Surveillance Assistants, nurses, and non-physician clinicians (clinical officers).^17^ All testing was conducted at the district hospital. Mchinji health-care providers have used the Lifebox^®^ oximeter and adult clip probe since 2012.^9,12^

#### Sylhet, Bangladesh.

In northeast Bangladesh, we conducted the study in collaboration with the research consortium Projahnmo in Sylhet. Physicians and nurses staff Projahnmo-supported clinics, and CHWs perform household surveillance. Projahnmo clinical staff and CHWs have used Masimo Rad5^®^ pulse oximeters (Irvine, CA) and reusable pediatric wrap probes since 2015.

#### London, UK.

In the United Kingdom, we conducted the study at the Great Ormond Street Hospital where pulse oximetry using single-use probes is routine. Health-care workers were highly trained nurses familiar with pulse oximetry, but not reusable probes, and not the Lifebox^®^ oximeter.

### Recruitment.

Study staff purposefully recruited HCWs with prior pulse oximetry experience, but without prior project involvement. In the LRS, this included CHWs. All HCWs received training that included an approximately 1 hour overview of pulse oximetry, orientation to the device, practice with using the device on other HCWs and volunteer children, and other protocol specifics (Supplemental Appendix 2). We reimbursed LRS HCWs for travel.

We recruited children aged < 5 years from inpatient and outpatient settings using convenience sampling and categorized them by age: neonates (0–28 days), infants (1–11 months), toddlers (12–23 months), and children (24–59 months). We excluded children who were clinically unstable, were receiving oxygen, or had an SpO_2_ < 95% on expert screening. Seven clinicians with extensive pediatric oximetry experience in LRS conducted participant screening and obtained reference pulse oximetry measurements (N. B., I. A. W., B. Z., A. A. M. H., S. C. S., K. M., E. D. M.).

### Sample size.

Using usability testing, we needed at least 15 users per study site to ensure we identified most device issues.^18,19^ For statistical analysis, we needed at least 292 measurements to test the probe against our pre-defined aspirational goal of 95% of measurements in < 1 minute with 2.5% precision. To allow for stratified analyses by site, end-user type, and age group, we aimed to recruit 17 HCW at each study site, with each testing 12 children divided equally by age categories (*N* = 204 per site). Experts recorded approximately the same number of measurements per site, also equally divided by age strata.

### Data collection.

 We used a Lifebox^®^ oximeter (version 1.5). To allow the expert to conduct reference SpO_2_ measurements, an independent observer recorded the child’s demographic data, condition, clinical features, and timed the expert SpO_2_ measurement (Supplemental Appendix 3). The observer was a member of the research team who was present to provide independent timing and data recording of the measurement process. Children were first screened by the experts, and those with an SpO_2_ > 95% were permitted to participate in HCW testing. For expert measurements, the expert and/or caregiver could distract the child and support the limb. The independent observer started a timer once probe placement was complete and stopped it when the expert said “stop,” signifying that, in the expert’s view, this was a successful SpO_2_ reading. Experts were trained to assume an SpO_2_ reading as reliable if the measurement had a consistent, high-amplitude plethysmography waveform, accompanied by an SpO_2_ and heart rate that, in their judgment, was biologically plausible for that child. The SpO_2_, heart rate, and additional observations were then recorded. If an SpO_2_ was not obtained at the first location, the expert could adjust the probe or use another location for a maximum of 5 minutes, at which point the testing was stopped. Biologically implausible measurements, for example, were measurements that had a normal SpO_2_ but had a heart rate lower than the approximate 10th centile for the age of the child,^20^ or measurements that had a severely abnormal SpO_2_ value in an otherwise clinically stable child.

Health-care workers were blinded to expert measurements. To allow the expert to observe HCW testing without distractions and ensure accurate timing, the independent observer also timed all HCW measurements (Supplemental Appendix 3). Health-care workers followed the exact same measurement process as completed by experts and used the same criteria for determining whether the SpO_2_ was reliable. The expert also determined whether the HCW reading was reliable in their judgment by using the same metrics used with the expert measurement, and the observer separately recorded this information without the HCW’s knowledge. The HCW could attempt to obtain an SpO_2_ for a maximum of 5 minutes, at which point the testing was stopped. No additional guidance or retraining was provided to HCWs by experts during or between measurements if the HCWs were taking measurements incorrectly. De-identified data were captured electronically and uploaded onto secure servers.

Following testing, HCWs completed a written usability questionnaire (Supplemental Appendix 4). Usability testing was self-completed in writing by each HCW after completing their SpO_2_ measurements, with assistance to clarify questions by the research team, as necessary.

### Data analysis.

The primary outcome was a successful measurement in < 1 minute; secondary outcomes were successful measurements in < 2 and < 5 minutes. A successful HCW SpO_2_ reading was defined to be relevant for real-world practice as being completed in 5 minutes or sooner, having a consistent, high-amplitude plethysmographic waveform, and displaying a value > 95% (clinically stable) or within ±2% of an immediately repeated expert measurement if the HCW SpO_2_ was < 95%. Expert readings were all assumed to be reliable, and therefore were considered successful if achieved in < 5 minutes.

We described the proportion of measurements in < 1, < 2, and < 5 minutes. We stratified results by the child’s age, end-user cadre, and study site; differences between the proportion of successful readings in < 1 minute were evaluated by using χ^2^ tests, and median time to reading by Kruskall–wallis tests. We used univariable and multivariable logistic regressions for predictors of successful measurements < 1 minute. We a priori selected the following predictors: child’s age, weight, ethnicity, measurement site and relocation, child’s condition, end- user cadre, and study site. Analyses were adjusted for clustering within children using robust standard errors. All analysis was conducted using Stata 14 (StataCorp LLC, College Station, TX).

Likert scale questions from usability questionnaires were described, primarily for HCW probe usability on age categories. Common themes from free-text responses about usability, challenges, and suggested improvements were coded.

### Ethics.

Ethical approval, including review of our consent forms and information sheets, was provided by Malawi (ref: 16/4/1570), Bangladesh (ref: BMRC/NREC/2013-2016/1272), University College London (ref: 8075/003), Johns Hopkins (ref: IRB00047406), and the London Dulwich committee (ref: 16/LO/2208). Authors can be contacted directly for any additional materials. Written consent was obtained from all HCWs and caregivers in the United Kingdom; verbal consent was obtained from caregivers in Malawi and Bangladesh. The study was registered on the ClinicalTrials.gov database (ref. NCT02941237).

## RESULTS

### Testing process.

**Figure 1.**
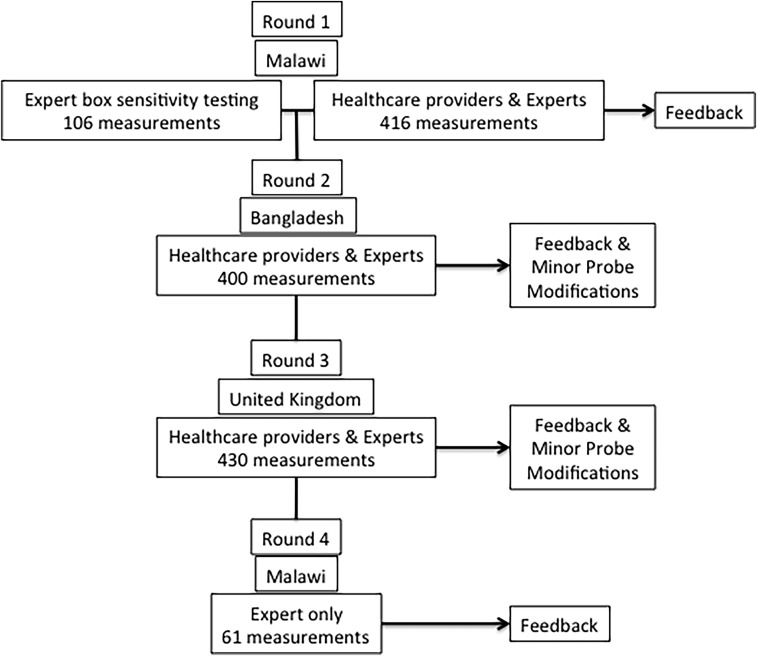
Usability testing flowchart.

We conducted four iterative testing rounds, with HCW and expert feedback between the first three rounds and expert feedback after the fourth round (Figure 1). We tested the same probe design in the first and second rounds. Another expert-only testing session was conducted in Malawi using a Nellcor^®^ box (Medtronic, Minneapolis, MN) to triangulate our results with a device that was compatible with the probe and incorporated motion sensitive software. This allowed us to discriminate between the oximeter and probe performance by controlling for the oximeter’s algorithmic design.

**Figure 2.**
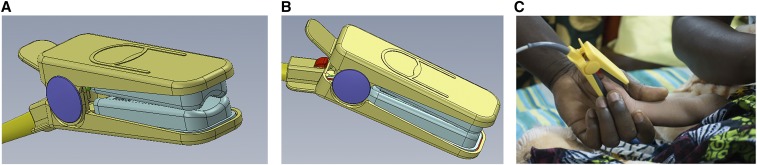
Final probe design (**A** and **B**) and during usability testing in Malawi (**C**).

Round 2 testing confirmed round one observations, so we refined the probe before round 3, adding a firmer pad beneath the light detector and a revised pivot to open the probe wider (Figure 2). Based on round 3 feedback, we shifted the light emitting diode and detector 5 mm away from the probe hinge, and changed the internal padding curvature. The fourth round of expert-only testing completed an LRS field performance check on the final probe design.

### Children and health-care provider characteristics.

Overall, 536 children participated (Table 1); 44.0% (236/536) were Malawian. Participants were a median of 11 months old (interquartile range [IQR]: 3, 25), weighed an average of 8.5 kg (standard deviation [SD], 4.0 kg), and had a median SpO_2_ of 97% (IQR: 95%, 98%). Overall, the most frequent primary diagnosis was acute respiratory infection (25%, *N* = 131), although 85% of UK participants had a noninfectious diagnosis or were healthy (128/150). A total of 51 HCWs participated. Community health workers were the most frequent LRS participants (55.8%, 19/34); all UK participants were nurses (100%, 17/17).

**Table 1:** Participant characteristics of pulse oximeter probe usability testing

Characteristic		Overall	Malawi	Bangladesh	United Kingdom
Children		*N* = 536	*N* = 236	*N* = 150	*N* = 150
Age in months, median (IQR)		11 (3–25)	10.5 (0–24)	12 (4–26)	13.5 (4–28)
Weight in kg, mean (SD)		8.5 (4.0)	8.3 (3.7)	7.9 (3.6)	9.4 (4.9)
SpO_2,_ median (IQR)		97 (95–98)	97 (95–98)	96 (90–98)	98 (97–98)
Primary diagnosis, *n* (%)	ARI	131 (25%)	62 (26%)	68 (45%)	1 (1%)
	Fever	57 (11%)	44 (19%)	12 (8%)	1 (1%)
	Other infectious	98 (19%)	46 (19%)	51 (34%)	1 (1%)
	Cardiac disease	13 (2%)	–	–	13 (9%)
	Other noninfectious	107 (20%)	9 (4%)	13 (9%)	85 (57%)
	New born	64 (12%)	55 (23%)	3 (2%)	6 (4%)
	Healthy	66 (12%)	20 (8%)	3 (2%)	43 (29%)
Health-care providers		*N* = 51	*N* = 17	*N* = 17	*N* = 17
Job title, *n* (%)	Physician	9 (18%)	–	9 (53%)	–
	Nurse	20 (39%)	3 (18%)	–	17 (100%)
	Non-physician clinician	3 (6%)	3 (18%)	–	–
	CHW	19 (37%)	11 (65%)	8 (47%)	–
Years working, median (IQR)		4 (1.5–10)	10 (5–10)	1 (1–2)	8 (4–20)

ARI = acute respiratory infection; CHW = community health worker; IQR = interquartile range; SD = standard deviation; SpO_2_ = peripheral oxygen saturation.

### SpO_2_ by HCW cadre.

A total of 1,307 SpO_2_ measurements were obtained (Table 2), and more than two-thirds (67%, 876/1,307) were achieved in < 1 minute, 81% (1,059/1,307) in < 2 minutes, and 90% (1,181/1,307) in < 5 minutes. The median time to a successful SpO_2_ was 29.8 seconds (IQR: 17.6, 61.5). We found a difference in the proportion of successful measurements achieved on the same patients in < 1, < 2, and < 5 minutes between non-CHWs, CHWs, and experts during the first two rounds. Experts achieved successful measurements in < 1 minute in 69% (282/409) versus 68% (155/228) and 59% (105/179) in CHWs and non-CHWs, respectively (*P* = 0.04). A similar trend was seen for < 2 minutes, with successful readings in 87% (357/409), 79% (180/228), and 69% (123/179; *P* < 0.01) for experts, CHWs, and non-CHWs, and also in < 5 minutes, with successful readings in 97% (experts, 398/409), 82% (CHWs, 187/228), and 75% (non-CHWs, 135/179, *P* < 0.01). Experts and non-CHWs took a longer median time to achieve successful measurements than CHWs (32.9 and 31.9 seconds versus 25.0 seconds, *P* < 0.01). In the United Kingdom, we found no difference in achieving a successful measurement between experts and HCWs.

**Table 2:** Results from pulse oximeter probe prototype testing in Malawi, Bangladesh, and the UK according to expert and HCW

Testing round		Total SpO_2_ tests	Quality SpO_2_ < 1 minute, *n* (%)	95% CI	Quality SpO_2_ < 2 minutes, *n* (%)	95% CI	Quality SpO_2_ < 5 minutes, *n* (%)	95% CI	Median time in seconds (IQR)
Cumulative	Overall	1,307	876 (67%)	64%, 70%	1,059 (81%)	79%, 83%	1,181 (90%)	89%, 92%	29.5 (17.6–61.5)
	Expert	689	470 (68%)	65%, 72%	584 (85%)	82%, 87%	660 (96%)	94%, 97%	32.0 (18.4–66.0)
	HCW	618	406 (66%)	62%, 69%	475 (77%)	73%, 80%	521 (84%)	81%, 87%	26.0 (16.8–55.4)
Round 1 (Malawi)	Overall	416	287 (69%)	64%, 73%	337 (81%)	77%, 85%	375 (90%)	87%, 93%	25.3 (16.1–55.3)
	Expert	211	147 (70%)	63%, 76%	176 (83%)	78%, 88%	200 (95%)	91%, 97%	24.8 (16.2–64.6)
	HCW	205	140 (68%)	61%, 75%	161 (79%)	72%, 84%	175 (85%)	80%, 90%	25.3 (15.6–49.9)
Round 2 (Bangladesh)	Overall	400	255 (64%)	59%, 68%	323 (81%)	77%, 84%	345 (86%)	82%, 89%	35.0 (20.6–60.8)
	Expert	198	135 (68%)	61%, 75%	181 (91%)	87%, 95%	198 (100%)	98%, 100%	42.0 (23.4–66.0)
	HCW	202	120 (59%)	52%, 66%	142 (70%)	63%, 77%	147 (73%)	66%, 79%	29.0 (19.0–48.0)
Round 3 (UK)	Overall	430	297 (69%)	64%, 73%	351 (82%)	78%, 85%	400 (93%)	90%, 95%	26.8 (15.4–62.4)
	Expert	219	151 (69%)	62%, 75%	179 (82%)	76%, 87%	201 (92%)	87%, 95%	27.6 (16.1–59.3)
	HCW	211	146 (69%)	62%, 75%	172 (82%)	76%, 87%	199 (94%)	90%, 97%	26.3 (14.7–64.2)
Round 4 (Malawi–expert only)	Overall	61	37 (61%)	47%, 73%	48 (79%)	66%, 88%	61 (100%)	94%, 100%	46.0 (22.0–100.0)

CI = confidence interval; HCW = health-care worker; IQR = interquartile range; SpO_2_ = peripheral oxyhemoglobin saturation; UK = United Kingdom.

A total of 5.8% (76/1,307) of all SpO_2_ measurements were biologically implausible (Supplemental Table 1). Notably, there was a greater proportion of biologically implausible readings by HCWs in both LRS than in the United Kingdom. Overall, HCWs provided 10.1% biologically implausible readings in Malawi (20/195), 27.2% in Bangladesh (55/202), and 0.5% in the United Kingdom (1/211, *P* < 0.001).

### Pulse oximeter box sensitivity testing.

A total of 106 measurements were taken using the Nellcor^®^ box, achieving a median time of 29.8 seconds and 67% in < 1 minute (Supplemental Table 2). There was no statistical difference between the median time to reading and round one Malawi expert readings (*P* = 0.32), but there was an upward trend in the proportion of successful measurements achieved < 5 minutes by Nellcor^®^ (94.8% versus 99.1%, *P* = 0.06).

### SpO_2_ by child’s age category.

Table 3 shows results by age. Overall, a higher proportion of measurements on toddlers and children were obtained in < 1, < 2, and < 5 minutes compared with neonates and infants. Measurements took the longest median time on neonates (50.1 second, IQR: 28.0, 98.4) and infants (41.3 seconds IQR: 21.9, 83.6), versus toddlers (23.2 seconds, IQR: 15.0, 44.7) and children (19.8 seconds, IQR: 13.9, 33.7; *P* < 0.01). This was consistent across all rounds.

**Table 3:** Results from pulse oximeter probe testing in Malawi, Bangladesh, and the UK according to child’s age category

Testing round		Total SpO_2_ tests	Quality SpO_2_ < 1 minute, *n* (%)	95% CI	Quality SpO_2_ < 2 minutes, *n* (%)	95% CI	Quality SpO_2_ < 5 minutes, *n* (%)	95% CI	Median time in seconds (IQR)
Cumulative	Overall	1,307	876 (67%)	64%, 70%	1,059 (81%)	79%, 83%	1,181 (90%)	89%, 92%	29.5 (17.6–61.5)
	Neonate	312	160 (51%)	46%, 57%	219 (70%)	65%, 75%	268 (86%)	82%, 90%	50.1 (28.0–98.4)
	Infant	350	192 (55%)	49%, 60%	253 (72%)	67%, 77%	301 (86%)	82%, 89%	41.3 (21.9–83.6)
	Toddler	317	245 (77%)	72%, 82%	283 (89%)	85%, 92%	296 (93%)	90%, 96%	23.2 (15.0–44.7)
	Child	328	279 (85%)	81%, 89%	304 (93%)	89%, 95%	316 (96%)	94%, 98%	19.8 (13.9–33.7)
Round 1 (Malawi)	Overall	416	287 (69%)	64%, 73%	337 (81%)	77%, 85%	375 (90%)	87%, 93%	25.3 (16.1–55.3)
	Neonate	103	57 (55%)	45%, 65%	73 (71%)	61%, 79%	86 (84%)	75%, 90%	42.5 (24.9–87.4)
	Infant	109	60 (55%)	45%, 65%	80 (73%)	64%, 81%	96 (88%)	80%, 93%	35.0 (19.5–81.2)
	Toddler	102	78 (76%)	67%, 84%	87 (85%)	77%, 92%	94 (92%)	85%, 97%	22.1 (13.7–44.3)
	Child	102	92 (90%)	83%, 95%	97 (95%)	89%, 98%	99 (97%)	92%, 99%	17.9 (12.4–25.8)
Round 2 (Bangladesh)	Overall	400	255 (64%)	59%, 68%	323 (81%)	77%, 84%	345 (86%)	82%, 89%	35.0 (20.6–60.8)
	Neonate	87	38 (44%)	33%, 55%	59 (68%)	57%, 77%	66 (76%)	65%, 84%	57.7 (36.1–86.6)
	Infant	110	64 (58%)	48%, 68%	81 (74%)	64%, 82%	92 (84%)	75%, 90%	41.0 (23.7–65.0)
	Toddler	98	70 (71%)	61%, 80%	89 (91%)	83%, 96%	89 (91%)	83%, 96%	31.3 (19.3–53.4)
	Child	105	83 (79%)	70%, 86%	94 (90%)	82%, 95%	98 (93%)	87%, 97%	22.3 (17.0–40.8)
Round 3 (UK)	Overall	430	297 (69%)	64%, 73%	351 (82%)	78%, 85%	400 (93%)	90%, 95%	26.8 (15.4–62.4)
	Neonate	106	61 (58%)	48%, 67%	77 (73%)	63%, 81%	100 (94%)	88%, 98%	47.4 (26.4–108.4)
	Infant	115	57 (50%)	40%, 59%	78 (68%)	58%, 76%	97 (84%)	76%, 90%	50.9 (23.1–110.9)
	Toddler	104	87 (84%)	75%, 90%	95 (91%)	84%, 96%	100 (96%)	90%, 99%	17.4 (13.0–28.7)
	Child	105	92 (88%)	80%, 93%	101 (96%)	91%, 99%	103 (98%)	93%, 100%	18.9 (11.0–32.3)
Round 4 (Malawi–expert only)	Overall	61	37 (61%)	47%, 73%	48 (79%)	66%, 88%	61 (100%)	94%, 100%	46.0 (22.0–100.0)
	Neonate	16	4 (25%)	7%, 52%	10 (63%)	35%, 85%	16 (100%)	79%, 100%	78.5 (56.5–149.0)
	Infant	16	11 (69%)	41%, 89%	14 (88%)	62%, 98%	16 (100%)	79%, 100%	42.5 (20.5–90.5)
	Toddler	13	10 (77%)	46%, 95%	12 (92%)	64%, 99%	13 (100%)	75%, 100%	32.0 (22.0–46.0)
	Child	16	12 (75%)	48%, 93%	12 (75%)	48%, 93%	16 (100%)	79%, 100%	31.0 (20.0–94.5)

CI = confidence interval; IQR = interquartile range; SpO_2_ = peripheral oxyhemoglobin saturation.

### Child’s behavioral state.

The child’s behavioral state had an important relationship with the time to a successful SpO_2_ (Supplemental Table 3). The median time to a successful SpO_2_ was shorter if the child was asleep (24.0 seconds, IQR: 16.7, 38.3) or calm (26.0 seconds, IQR: 16.2, 55.7) rather than agitated (72.0 seconds, IQR: 30.3, 124.0) or crying (65.5 seconds, IQR: 36.2, 137.7; *P* < 0.01).

### Predictors of successful measurements.

Results from univariate and multivariate analysis of factors associated with successful measurements < 1 minute are in Table 4. In the adjusted model, increasing age and being asleep were associated with achieving an SpO_2_ in < 1 minute. The child being agitated or crying, repositioning the probe and first placing the probe across the foot, were all associated with failing to measure an SpO_2_ in < 1 minute.

**Table 4:** Factors associated with an SpO_2_ measurement achieved in ≤ 1 minute

Characteristic		SpO_2_ ≤ 1 minute	SpO_2_ > 1 minute	OR (95% CI)	*P* value	aOR (95% CI)	*P* value
Age	Neonate	160	152	1.00	–	1.00	–
	Infant	192	158	1.15 (0.85, 1.57)	0.35	1.87 (1.16, 3.02)	0.01
	Toddler	245	72	3.23 (2.29, 4.56)	< 0.01	4.33 (2.36, 7.97)	< 0.01
	Child	279	49	5.41 (3.71, 7.88)	< 0.01	3.90 (1.73, 8.81)	< 0.01
Weight	< 10 kg	486	354	1.00	–	1.00	–
	≥ 10 kg	375	71	3.85 (2.88, 5.13)	< 0.01	1.80 (0.90, 3.59)	0.09
Ethnicity	Black	349	161	1.00	–	1.00	–
	White	226	104	1.00 (0.74, 1.35)	0.98	1.08 (0.65, 1.82)	0.76
	Asian	290	164	0.82 (0.62, 1.07)	0.13	0.68 (0.44, 1.05)	0.08
	Other	11	2	2.54 (0.56, 11.58.)	0.22	5.98 (0.97, 36.77)	0.05
Site of first measure	Toe	793	319	1.00	–	1.00	–
	Foot	59	73	0.33 (0.23, 0.47)	< 0.01	0.30 (0.17, 0.53)	< 0.01
	Hand/finger	24	10	0.97 (0.46, 2.04)	0.92	0.56 (0.17, 1.85)	0.33
Number of probe repositions	None	832	167	1.00	–	1.00	–
	1 reposition	41	120	0.07 (0.05, 0.10)	< 0.01	0.06 (0.04, 0.11)	< 0.01
	≥ 2 repositions	3	115	0.01 (0.00, 0.02)	< 0.01	0.01 (0.00, 0.02)	< 0.01
Child’s condition	Calm	556	205	1.00	–	1.00	–
	Agitated	53	106	0.18 (0.13, 0.27)	< 0.01	0.26 (0.14, 0.47)	< 0.01
	Crying	34	75	0.17 (0.11, 0.26)	< 0.01	0.15 (0.07, 0.30)	< 0.01
	Sleeping	233	45	1.91 (1.34, 2.73)	< 0.01	3.53 (1.89, 6.58)	< 0.01
Tester	Non-CHW	251	139	1.00	–	1.00	–
	CHW	155	73	1.18 (0.83, 1.66)	0.36	0.76 (0.41, 1.42)	0.39
	Expert	470	219	1.18 (0.91, 1.53)	0.19	0.94 (0.63, 1.42)	0.78
Study site*	Malawi	324	153	1.00	–	–	–
	Bangladesh	255	145	0.83 (0.63, 1.10)	0.19		
	UK	297	133	1.05 (0.80, 1.40)	0.71		

aOR = adjusted odds ratio; CHW = community health worker; CI = confidence interval; kg = kilogram; OR = odds ratio; SpO_2_ = peripheral arterial oxyhemoglobin saturation; UK = United Kingdom.

* Excluded for collinearity with ethnicity.

### Health-care worker feedback.

**Figure 3.**
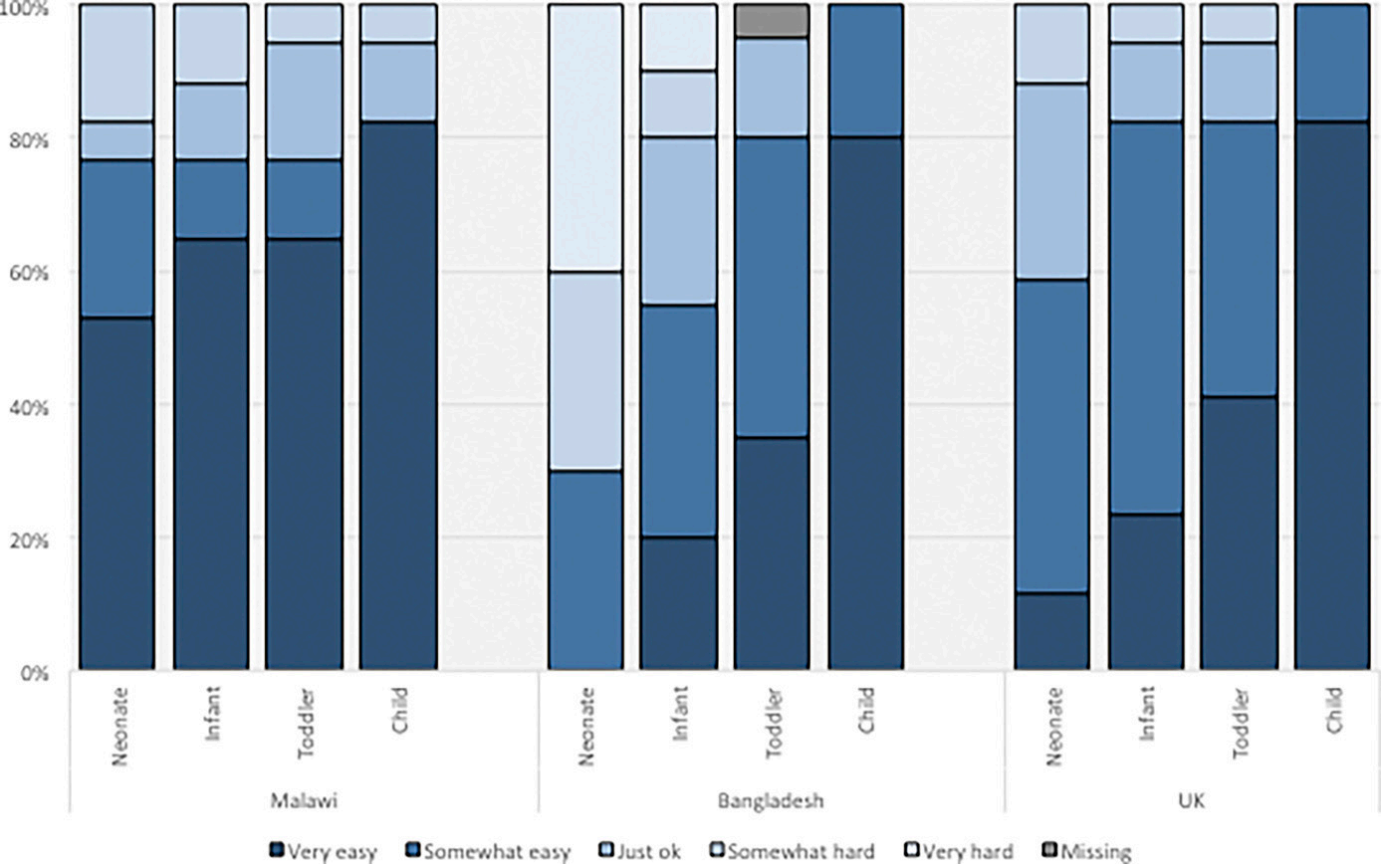
Feedback from health-care worker usability questionnaire from Malawi, Bangladesh, and the United Kingdom (UK). Answers in response to the question: “How easy did you find the probe to use in XX?” presented for the different age categories. This figure appears in color at www.ajtmh.org.

All 51 HCWs completed the questionnaire. Overall, 74% of HCWs either strongly or somewhat agreed that the probe was easy to use on all children aged 0–59 months. There was an upward trend in ease of use, with increasing age across all sites (Figure 3), but there were differences in responses between the sites. Eighty-eight percent of respondents strongly or somewhat agreed that the pulse oximeter would make their jobs easier, and 90% agreed that it would help them diagnose pneumonia. The main challenges raised by participants were the probe’s size relative to neonates, and readings during movement, with only 38% of respondents agreeing that it is easy to get a reading in a moving child. Additional HCW feedback is reported in Supplemental Table 4.

## DISCUSSION

We used a HCD process to design a novel pulse oximeter probe for use by HCWs, and evaluated the usability of the probe on children < 5 years old in low- and high-resource environments. Our primary outcome was the time to a successful SpO_2_ measurement. We achieved this 67% of the time in < 1 minute, 81% in < 2 minutes, and 90% in < 5 minutes, although we identified differences across testing rounds, with different user cadres and in different ages. In the final round, when all probe modifications were included, experts achieved a reading in < 5 minutes in 100%, < 2 minutes in 79%, and < 1 minute in 61% of children. Although these results suggest feasibility for use of this probe in LRS, and therefore support LRS implementation, it is important to acknowledge that they were lower than our a priori aspirational target of 95% of readings in < 1 minute. Notably, this target was more ambitious than an expert-developed TPP which set < 2 minutes as the ideal and < 5 minutes as the minimum performance. In retrospect, our study target was too ambitious for a lower cost device designed for newborns to 5 year olds, but meets the wider clinically acceptable minimum performance, even in a device unequipped with more sophisticated motion tolerant technology. We believe that achieving a time to measurement target of > 90% of measurements in < 1 minute is necessary for optimal implementation of pulse oximetry in LRS with high patient volumes and few health-care providers. Additional investments in pulse oximetry development are needed to meet this target. We are unaware of any other commercial pulse oximeter that has quantified end-user oximetry usability across the pediatric age spectrum by a range of end-users.

Through expert and end-user engagement, our HCD process established time to a successful SpO_2_ measurement as this study’s primary endpoint for usability testing. Consistently recording a successful SpO_2_ quickly is critical for feasibility of routine pulse oximetry screening of children in busy, understaffed LRS. Despite this, limited published data have examined SpO_2_ measurement times in LRS. A study from Malawi in children < 5 years old, using the Lifebox^®^ oximeter (version 1.0) and an adult clip probe, found that only 45% of HCW’s reported that on average SpO_2_ measurements took < 2 minutes.^9^ Here, we achieved 81% of measurements in < 2 minutes, suggesting that both the redesigned probe and previous box microprocessor upgrades have markedly improved performance. Emdin et al.^21^ explored HCW oximetry testing on infants < 60 days old in Pakistan, reporting readings in 94.4% in < 1 minute and 99% in < 5 minutes of infants. There are two likely explanations for the Pakistan study’s higher proportion of readings in < 1 minute. First, they used a pulse oximeter monitor and reusable probe with motion and low perfusion technology (Rad-5v^®^ and LNCS^®^ Y-I multisite sensor, Masimo^®^, > $700 USD; Irvine, CA,). However, this is prohibitively expensive technology for wide-scale LRS implementation. The Lifebox^®^ box and redesigned probe is expected to cost between $100 and $200/unit. Second, the Rad-5v^®^ does not display a plethysmography waveform. As a result, Emdin et al. may have recorded faster measurement times based on a less precise definition for reliable, successful SpO_2_ measurements.^22^ We recommend that for future pulse oximetry usability testing in LRS investigators consider the metric of time to a successful SpO_2_ measurement as the reference standard from which oximeter usability is evaluated.

Our study highlighted the factors associated with longer measurement times as a proxy for difficult SpO_2_ measurements. We found that HCWs took longer to achieve an SpO_2_ in < 1 year olds than in older children. In addition, we found that it took longer to achieve an SpO_2_ if the child was agitated or crying than when sleeping or calm. These findings were supported by HCW feedback and previous studies noting patient age and cooperation as associated with successful measurements.^9,22,23^ It is key that these factors are considered when developing future LRS pulse oximeters. Our findings suggest that low-cost motion tolerant technological innovation is essential for future devices.

In both Malawi and Bangladesh, there was a trend that CHWs performed better than non-CHWs who have more extensive training and education. Our previous focus group work suggests that CHWs place a higher “value” on pulse oximetry, which may lead to more careful adherence to SpO_2_ protocols, and highlights the enormous potential of community-based implementation programs for pulse oximetry.^9,24^

We also found that experts consistently performed better than HCWs in LRS testing, a difference not seen in the United Kingdom. LRS HCWs reported a higher proportion of biologically implausible measurements (18.9%) than UK HCWs (0.5%). Several reasons may account for these findings. First, LRS HCWs are likely to be less familiar with the science behind pulse oximetry and the interpretation of SpO_2_ readings in the broader clinical context. This lack of clinical education and training may lead to poorer comprehension of the biological plausibility of SpO_2_ readings, and was probably exacerbated in Bangladesh where HCWs were less familiar with the Lifebox^®^ and had been using oximetry for a shorter period of time than in Malawi. Second, LRS HCWs without pediatric training may be less adept at applying techniques with children to reduce movement and agitation to achieve a successful SpO_2_. This includes correct probe placement, appropriate support of the limb, or other distraction techniques including using toys or breastfeeding. Finally, LRS HCWs may have felt obligated to report an SpO_2_ to experts even if they believed that the SpO_2_ was biologically implausible. These differences could potentially be addressed with improved education, rather than basic task-specific training and ongoing mentorship approaches, a critical consideration for wide-scale implementation of pulse oximetry in LRS.

This study had several limitations. The act of directly observing HCWs obtaining oximetry readings may have caused them to change their usual practice, the Hawthorne effect, altering the final measurements HCWs provided.^25^ Because the HCWs were aware that their measurement time was being recorded, this could have led to hastier, inaccurate readings, thinking a faster time was more important than what we defined as a successful measurement. This may be more pronounced in LRS and may therefore, in part, account for the higher proportion of lower successful readings provided by LRS HCWs. We did anticipate this potential bias before the study, and during trainings, we stressed the need for HCWs to believe the SpO_2_ to be true. There were also notable differences between patient populations across settings. For example, more LRS children had infectious diagnoses and more UK children had chronic illnesses. United Kingdom children with chronic illnesses may be more familiar with pulse oximetry, and therefore more compliant with measurements. Finally, because of the convenience sample design and patient availability, we re-tested 332 of 526 children; repeat testing may have led to bias if the experts, HCWs, or children modified their behavior between measurements. To account for this, we adjusted for clustering within children in the regression analysis.

In conclusion, this HCD usability study indicates that, in children < 5 years old, it is possible to use a pulse oximetry probe to achieve successful SpO_2_ readings in 67% of children in < 1 minute, 81% in < 2 minutes, and 90% in < 5 minutes. These results are encouraging for an innovative pediatric pulse oximetry probe that is reusable and low cost. We believe that this design is an appropriate “universal probe” suitable for use by LRS HCWs on patients of any age, including newborns. Our findings highlight the factors associated with longer measurement times, in particular movement artifact, and suggest that task-specific training is sufficient for LRS study settings, but enhanced training and ongoing supervision is still likely necessary to successfully and sustainably implement pulse oximetry in a non-study LRS settings. We recommend future pulse oximetry usability testing studies in LRS to use a HCD process that incorporates feedback from field experts and end-users. We additionally recommend future usability studies to use time to a successful SpO_2_ measurement as the standard for assessing implementation feasibility of oximeter devices in LRS. Next steps could focus on developing low-cost LRS pediatric pulse oximeters as specialized spot-check devices with high motion tolerance that display the most reliable, single SpO_2_ reading for easier HCW interpretation.

## Supplementary Material

Supplemental appendices and tables
