# Supplementary material for: Usability Testing of a Reusable Pulse Oximeter Probe Developed for Health-Care Workers Caring for Children < 5 Years Old in Low-Resource Settings
Source: Am J Trop Med Hyg. 2018 Aug 20;99(4):1096–104. doi: 10.4269/ajtmh.18-0016 (PMC6159595; doi:10.4269/ajtmh.18-0016)
Supplement: Supplementary file 1 [file tpmd180016.SD1.pdf]

Supplemental table 1. Stratification of unsuccessful SpO_2 readings by time and biological implausibility.

Testing round		Total SpO_2 tests	Unsuccessful (>5 minutes)	Unsuccessful (biologically implausible)
Cumulative	Overall	1307	50 (4%)	76 (6%)
	Expert	689	29 (4%)	-
	HCW	618	21 (3%)	76 (12%)
Round 1 (Malawi)	Overall	416	21 (5%)	20 (5%)
	Expert	211	11 (5%)	-
	HCW	205	10 (5%)	20 (10%)
Round 2 (Bangladesh)	Overall	400	0	55 (14%)
	Expert	198	0	-
	HCW	202	0	55 (27%)
Round 3 (United Kingdom)	Overall	430	29 (7%)	1 (0.2%)
	Expert	219	18 (8%)	-
	HCW	211	11 (5%)	1 (0.5%)
Round 4 (Malawi – expert only)	Overall	61	0	-

SpO_2 indicates non-invasive arterial oxyhemoglobin saturation.
