# Supplementary material for: Usability Testing of a Reusable Pulse Oximeter Probe Developed for Health-Care Workers Caring for Children < 5 Years Old in Low-Resource Settings
Source: Am J Trop Med Hyg. 2018 Aug 20;99(4):1096–104. doi: 10.4269/ajtmh.18-0016 (PMC6159595; doi:10.4269/ajtmh.18-0016)
Supplement: Supplementary file 2 [file tpmd180016.SD2.pdf]

Supplemental Table 2: Pulse oximeter box sensitivity testing comparing Nellcor with Lifebox

	Total SpO_2 tests	SpO_2 <1 minutes n (%; 95% CI)	SpO_2 <2 minutes n (%; 95% CI)	SpO_2 <5 minutes n (%; 95% CI)	Median time in seconds (IQR)
Overall: Nellcor	106	71 (67%; 57, 76)	89 (84%; 76, 90)	105 (99%; 95, 100)	29.8 (20, 80.1)
Neonate	26	15 (58%; 37, 77)	23 (88%; 70, 98)	26 (100% 87, 100)	38.5 (21.7, 84.7)
Infant	26	12 (46%; 27, 67)	16 (62%; 41, 80)	25 (96%; 80, 100)	60.2 (23.3, 161.8)
Toddler	26	18 (69%; 48, 86)	22 (85%; 65, 96)	26 (100%; 87, 100)	24.7 (19.6, 89.2)
Child	28	26 (93%; 76, 99)	28 (100%; 88, 100)	28 (100%; 88, 100)	20.2 (18.4, 30.3)
Overall: Lifebox*	211	147 (70%; 63, 76)	176 (83%; 78, 88)	200 (95%; 91, 97)	24.8 (16.2, 64.6)
Neonate	52	30 (58%; 43, 71)	42 (81%, 67, 90)	50 (96%; 87, 100)	44.7 (27.6, 98.7)
Infant	57	31 (54%; 41, 68)	41 (72%; 58, 83)	51 (89%; 78, 96)	30.0 (19.8, 100.0)
Toddler	51	38 (75%; 60, 86)	43 (84%; 71, 93)	49 (96%; 87, 100)	23.4 (15.0, 56.5)
Child	51	48 (94%; 84, 99)	50 (98%; 90, 100)	50 (98%; 90, 100)	16.6 (12.3, 23.2)

SpO_2 indicates non-invasive oxyhemoglobin saturation; CI, confidence interval; IQR, interquartile range.

*Note: this includes Round 1 expert measurements from Malawi only as the comparison group.

Supplemental Table 3: Results from pulse oximeter probe usability testing in Malawi, Bangladesh, and the United Kingdom according to child's behavioural state

	Total SpO_2 tests	SpO_2 <1 minutes n (%; 95% CI)	SpO_2 <2 minutes n (%; 95% CI)	SpO_2 <5 minutes n (%; 95% CI)	Median time in seconds (IQR)
Calm	761	556 (73%; 70, 76)	656 (86%; 84, 89)	718 (94%; 92, 96)	26.0 (16.2, 55.7)
Agitated	159	53 (33%; 26, 41)	90 (57%; 49, 64)	122 (77%; 69, 83)	72.0 (30.3, 124.0)
Crying	109	34 (32%; 23, 41)	59 (55%; 44, 64)	80 (73%; 64, 81)	65.5 (36.2, 137.7)
Sleeping	278	233 (84%; 79, 88)	254 (91%; 87, 94)	261 (94%; 90, 96)	24.0 (16.7, 38.3)

SpO_2 indicates non-invasive oxyhemoglobin saturation; CI, confidence interval; IQR, interquartile range.
