# Supplementary material for: Usability Testing of a Reusable Pulse Oximeter Probe Developed for Health-Care Workers Caring for Children < 5 Years Old in Low-Resource Settings
Source: Am J Trop Med Hyg. 2018 Aug 20;99(4):1096–104. doi: 10.4269/ajtmh.18-0016 (PMC6159595; doi:10.4269/ajtmh.18-0016)
Supplement: Supplementary file 3 [file tpmd180016.SD3.pdf]

Supplemental table 4.

**Task Completion:**	Very easy	Somewhat easy	Just ok	Somewhat hard	Very hard	Missing
How easy did you find it to switch on the oximeter?	48 (94%)	1 (2%)	2 (4%)	-	-	-
How easy did you find attaching the probe to the oximeter?	39 (76%)	9 (18%)	3 (6%)	-	-	-
How easy did you find it to place the probe on children of different ages:						
Neonates (0-1 months)?	11 (22%)	18 (35%)	6 (12%)	11 (22%)	5 (10%)	-
Infants? (2-11 months)	19 (37%)	19 (37%)	7 (14%)	4 (8%)	2 (4%)	-
Young children? (12-23 months)	24 (48%)	16 (32%)	8 (16%)	2 (4%)	-	-
Older children? (24-59 months)	41 (80%)	7 (14%)	2 (4%)	1 (2%)	-	-
How did you find it deciding whether the reading was reliable?	25 (49%)	16 (31%)	4 (8%)	5 (10%)	-	1 (2%)
**Probe specific:**	Strongly agree	Somewhat agree	Neither agree or disagree	Somewhat disagree	Strongly disagree	Missing
The probe is easy to use on all children 0-59 months	19 (37%)	20 (39%)	3 (6%)	6 (12%)	3 (6%)	-
The probe does not fall off the child easily	30 (59%)	14 (27%)	1 (2%)	3 (6%)	3 (6%)	-
It is easy to check to see if the probe is working	33 (65%)	13 (25%)	1 (2%)	1 (2%)	3 (6%)	-
It is easy to use the probe, even if the child is moving	5 (10%)	15 (29%)	1 (2%)	10 (20%)	19 (37%)	1 (2%)
The probe does not hurt the child	41 (80%)	8 (16%)	1 (2%)	-	-	1 (2%)
The probe will not break easily	32 (63%)	12 (24%)	3 (6%)	2 (4%)	-	2 (4%)
The probe will be easy to keep clean	36 (71%)	12 (24%)	1 (2%)	-	2 (4%)	-
**Oximeter and the probe:**	Strongly agree	Somewhat agree	Neither agree or disagree	Somewhat disagree	Strongly disagree	Missing
I found it easy to learn to use this oximeter and probe	37 (73%)	14 (27%)	-	-	-	-
The oximeter is quick to give an oxygen saturation result	21 (41%)	15 (29%)	5 (10%)	4 (8%)	5 (10%)	1 (2%)
The oximeter and probe are easy to carry and to store	44 (86%)	6 (12%)	-	-	-	1 (2%)
The oximeter and probe will not hurt the child if I leave it with the child	38 (75%)	8 (16%)	2 (4%)	-	2 (4%)	1 (2%)
It is possible to use the oximeter if the light is poor, or if there is no light	30 (59%)	11 (22%)	4 (8%)	-	4 (8%)	2 (4%)
It is possible to use the oximeter and probe in bright sunlight	16 (31%)	10 (20%)	11 (22%)	-	1 (2%)	13 (25%)*
This oximeter and probe will help me to diagnose pneumonia in children	26 (51%)	17 (33%)	2 (4%)	2 (4%)	1 (2%)	3 (6%)
This oximeter and probe will make my job easier	27 (53%)	16 (31%)	3 (6%)	2 (4%)	1 (2%)	2 (4%)

*This was not answered by many healthcare workers who only used the probe inside in well-lit hospital wards.
